# Hôtel Dieu

**Published:** 1829-10-01

**Authors:** 


### XVII.

### HOTEL DIEU.

### LIGATURE OF THE ARTERY BEYOND THE ANEURISMAL TUMOUR.*

We have no intention of re-arguing this question, nor raking up the discords and dissentions of which it has been the signal, if not a material cause. Throughout the whole of the discussions which must still be fresh in the memory of our readers, we saw no occasion to change the opinion we expressed at their very first blush. We believed then, and believe still, that the operation ultra aneurysma is applicable to the common carotid, and to that only, because no branches intervene between the site of the ligature and the sac; in other words, because the circulation is no longer kept up through the latter. This is the conclusion which reasoning, a priori, would and did lead us to adopt, and the *facts* that have hitherto occurred have not tended to disprove its truth. On this point, however, the opinions of the profession in this country are still unsettled, and we question whether all the cases of Mr. Wardrop have contributed here, where they are best appreciated, to decide the matter either one way or other. In France, however, and indeed on the Continent generally, the garbled statements and noisy fanfaronade of a certain organ now almost defunct, made more impression than they did at home, and the brilliant issue of the late operations passed current through all the journals and salons. As usually happens, our foreign brethren conceived we were as much delighted as themselves, and have taken up the inquiry with the most furious zeal, just when we were beginning to view it in a sober and rational way. Dupuytren has recently performed the operation at the Hôtel Dieu, and the interest it seems to have excited in Paris, at least if we may judge by the medical press, has been of the most intense description. Certainly we know no more favourable circumstances for determining the real value of an operation, than those under which the present has been placed. Performed before hundreds of spectators of almost every European nation, by a surgeon of high and undoubted eminence, both the patient and the public have justice done them. Here there can be no concealment, no smothering of the facts, no sneaking equivocations, nor reckless mis-statements. What is done can be seen, what follows *must* be known; science is not vitiated, nor the profession (to use a mild term) deceived; in short, it is as fairly as one case can be so, an experimentum crucis. We shall proceed, without further preamble, to the details, which assume, on the foregoing accounts, a great and permanent importance.

The patient, a country labourer, forty years of age, and of good constitution, was admitted into the Hôtel Dieu on the 28th of May of the present year. Five months previously he began to suffer from pain in the whole of the right upper extremity, accompanied, after some time, by difficulty of motion, feebleness, and swelling. The next thing observed was pain just above the right clavicle, where a tumour, the size of a nut, appeared, and gradually augmented in volume, whilst the weakness and swelling of the arm increased pari passu. The patient now consulted a medical

---

* Journal Hebdom. No. 38.

man, who informed him that he laboured under aneurism of the subclavian, and recommended perfect rest, and the application of ice to the tumour. These means, however, were productive of no good effect, for the disease became aggravated every day, and the following was the state upon admission. The tumour was as large as one's fist, and extended from the median line of the neck and sterno-clavicular joint, to near the scapulo-humeral articulation. Mounting upwards towards the trapezius muscle, it formed a considerable prominence, and extended backwards even into the supra-spinatous fossa. The pulsations, which corresponded with those of the heart, were extensive and powerful; the integuments were sound, the whole of the limb much swollen and œdematous, and affected with severe shooting pains, but neither altered in temperature or hue. No tumour was distinctly perceived beneath the clavicle, but M. Sanson, on close examination, imagined that he felt in that direction a prolongation of the aneurism, an idea which subsequently proved to be correct. The common carotid could be traced unaffected with disease. The general health of the patient was good, there was scarcely any cough, and the respiration was unaffected. The heart, when examined by auscultation, presented no unusual bruit, but the pulsation of the ventricles, was strong and sonorous, and heard over a pretty considerable space. It was difficult to determine the precise portion of the subclavian artery affected with the disease, or to pronounce that it had or had not extended to the innominata.

The patient was twice bled and compresses dipped in a lotion of acetate of lead were applied to the tumour, which was also covered with a bladder of pounded ice. The disease, however, perceptibly increased, especially in the direction backwards, and it now became a very serious question, what remained for the surgeon to attempt? The Valsalvan treatment promised little, and indeed, in M. Dupuytren's experience, it had often been productive of ill instead of good. The uncertain extent of the disease towards the heart, and the probable implication of the innominata, were powerful reasons against applying a ligature in this situation; and indeed, had the innominata been proved to be sound, M. Dupuytren did not think the event of the two operations on that vessel, already performed by Mott and Graefe, sufficiently favourable to justify a third attempt. In short, it was agreed on to apply the ligature beyond the tumour, and on the 12th of June the operation was performed in the theatre of the Hôtel Dieu, before a great number of spectators.

The patient was placed upon his back in a bed, properly supported by assistants, and the right arm was separated from the side. An incision through the skin and cellular membrane, about three inches long, was then commenced near the sterno-clavicular articulation, and carried about two fingers' breadth below the clavicle in the direction of the fibres of the deltoid, and a little transversely to those of the pectoralis major. The innermost fibres of the former muscle and those of the latter were next divided, and the different layers of fascia and cellular membrane in front of the axillary artery were also cut through. The pectoralis minor was divided for about three quarters of its breadth, and the axillary vein, which was greatly swollen, and almost entirely obscured the artery, was exposed throughout its whole extent. The separation of the artery and vein was only accomplished by a cautious and slow dissection, when a silk ligature was passed round the former. In order to ascertain that all was right, M. Dupuytren took hold of the ligature at either end, and by lifting it up, and pressing at the same time the artery on it with his finger, was able to arrest or permit at pleasure the circulation through the limb. This, of course, decided that the artery had been found, and the absence of all pain also shewing that no nerve was included, the ligature was tied at once. At the instant that this was done, M. Sanson, who had his finger placed upon the tumour, perceived that about twenty irregular and precipitate pulsations occurred all at once, after which they resumed their former ratio. The wound was lightly dressed, and compresses dipped in a very weak solution of the acetate of lead were continued as a local application, together with the bag of pounded ice over all. During the operation, no less than 14 vessels were secured with ligatures, and M. Dupuytren ascertained by examination with his finger that M. Sanson had been right in thinking that a portion of the aneurism extended down underneath the clavicle.

In the course of the day the patient experienced dyspnœa and general malaise, for which he was bled from the arm, till a syncope of short duration was procured. On the 13th, the tumour had perceptibly diminished in size, but the pulsations were the same, and the limb was in statu quo. Demulcents and anodynes were ordered, and the patient took half a grain of the acetate of lead in distilled water. On the third day after the operation the patient was doing well, but the tumour, though "notably" diminished in size, *pulsated as strongly and superficially as ever*. Thus matters went till the 17th, (fifth day,) when the patient was restless, and the tumour was much disturbed by painful attacks of cough; the pulse too was strong and quick. M. Dupuytren prescribed a bleeding from the arm, but in the middle of the day it was found that the dressings were stained with scarlet blood, though the point of the wound from which the hæmorrhage proceeded could not be discovered. The wound was washed with cold water and the bleeding ceased. Venesection, however, was again employed. The blood lost scarce amounted to five or six ounces, and M. Dupuytren imagined that it might proceed from laceration of that portion of the aneurism which extended beneath the clavicle.

The patient passed the night of the 17th well, and all went on favourably till the 19th, when the bottom of the wound appeared tumid, as if the aneurismal tumour was extending in that direction. The patient had been bled on the 18th, and he was bled again to-day; the pulsations in the aneurism still continued unabated. On the morning of the 20th, little alteration was perceived—the swelling of the limb was much the same, and its temperature unaffected—the debility, as it had been since the 17th, considerable. Towards evening he complained of general malaise, swooning fits succeeded, and he died in the course of the night, without presenting any other remarkable symptom.

*Dissection.* The right arm was livid, engorged, traversed with numerous dark veins and its cuticle raised in several places. This condition had, according to the report of the "interne," occurred since the patient's death.

The cavities of the head and abdomen presented nothing unusual.

The first and second ribs upon which the tumour rested were worn, and in one point completely destroyed. Both pleural cavities contained about six or eight ounces of deep-coloured sero-sanguineous effusion. The heart was pale, flabby, and dilated in its cavities, with rather attenuated walls. That portion of the pleura covering the posterior part of the right lung was inflamed, with thin and recent false membranes on its surface, and the lung itself was in several points hepatized. The aorta, from its origin to three fingers' breadth above the point where it passes between the crura of the diaphragm, was so enormously dilated, as to admit the fist of a child, ten years of age to be placed in it. Its coats were very thick, and the inner was of livid red colour, and presented a number of "fungosities," and erosions, through which protruded spiculæ of osseous matter. The innominata was sound, but double its natural size.

The aneurismal tumour was exclusively confined to the subclavian, which it occupied, from its origin to its termination. It extended beneath the clavicle and behind the axillary artery, which was flattened over it at this point; and passed backwards to the suprà spinatous fossa. No rupture could be found in it to explain the hæmorrhage, which appears to have greatly surprised those concerned. The axillary artery was not divided by the ligature, and the vein and vessels, though greatly compressed by the tumour, had been fairly avoided. One of the latter was confounded with the parietes of the tumour, which were partly formed at the expense of the neighbouring parts.

So much for this important case, the result of which was looked to with a beating heart, no doubt, by several individuals in this country. The interest it appears to have excited in France was extreme, and the ridiculous things which were said about it, just what one expects from our lively neighbours. In the Lancette Française, a kind of imitation of our own precious exotic, the operation is described in much the same style as a modern Russian bulletin, and the astounding genius of M. Dupuytren is celebrated with all the arts of pen and ink!

"Il opére," says the French Editor, "il *réussit*. Honneur au génie," &c. &c. Of the operation, unfortunately, no one can entertain a doubt, but its *success* may be rather a matter of dispute, as the patient died on the eighth day after its performance. The Editor of the Parisian Lancet, like his very respectable confrère in England, will have found, by this time, that your thick and thin panegyrists, like your thick and thin libellers, occasionally cut a foolish figure.

Dropping, however, these remarks, let us look to the case itself. It appears to us that, à priori, it makes against the operation, because the patient died much more rapidly after its performance, than, in all probability, he would have done without it. Some of the French Journalists, led away by enthusiasm, or blinded by the reputed *amour propre* of their nation, have boldly asserted, that the operation itself was not the cause of death. We shall not waste words by arguing that question, for any unprejudiced man, on perusing the details of the case, must inevitably arrive at the opposite conclusion. An operation, however, may prove fatal, and yet, under the circumstances, be perfectly justifiable and proper for all that. Let us see if the present was in this predicament. The subclavian artery presents an aneurismal tumour — the axillary trunk is tied — secondary hæmorrhage occurs, though to no great amount— and, on the eighth day the patient sinks. On dissection the aneurism is found to be confined to the subclavian itself, but the innominata is greatly dilated, the aorta even more so, and extensively diseased through the whole of its thoracic portion, and the heart affected with passive aneurism. With such a combination of serious diseases of the vascular system, the fatal result is little to be wondered at. M. Dupuytren, however, is not discouraged, for he is made to say that, if another case presented itself, he would operate again in a similar way. For our own parts we see little encouragement to proceed, in any one part of this case. Not only did the operation prove the cause of death, but it failed, so far as in the trifling interval between its performance and the fatal issue it could do so, in abating the disease. The tumour, we are told, was diminished in size, but it pulsated as strongly as ever to the last; and in the notes of the dissection, not so much as a syllable is said of any deposition of recent coagulum within it. The account, indeed, of this part of the examination is singularly bad, for no mention is made of the nature of the aneurism, whether false or true, circumstances of considerable moment in forming an estimate of the case.*

We need scarcely tell our readers that we have deprecated through good and through bad report, the application of this method of operating to cases of aneurism of the subclavian and innominata. We objected to it first upon principle, we object to it now from experience. Aneurisms of the innominata and its two great branches at their origin, are seldom or never uncombined with disease of the aorta and heart, and what can an operation promise in such a state of things but disastrous and fatal results! DILATATIONS of these vessels are so common in elderly persons, especially females, that we question whether they shorten existence in any material degree. At all events, we have seen them remain for many years, and pro-

* In some of the other journals, we find it stated that the aneurism was a false one, formed by the destruction, more or less, of the arterial tunics.

duce no more inconveniences at the end of that period than at the beginning. The proper case for the "operation of Brasdor," is *aneurism* of the common carotid, for no vessels arise from that trunk between the ligature and sac. To expect, however, that success will follow this operation on the subclavian, when the circulation through the tumour is kept up by so many large branches, seems to us to be surgery run mad.

---

XVIII.

### HOTEL DIEU.

I. SUCCESSFUL EXTIRPATION OF THE UTERUS. BY PROFESSOR RECAMIER.*

We are happy to record another successful issue to this most formidable operation—and that in a public institution, where no colouring can be given to the facts—no veil of concealment thrown over the ultimate event.

*Case.* Agathy Pelagie, aged 50 years, was received into the Hôtel Dieu, on the 24th July, 1829, under M. Recamier. She had begun to menstruate at the age of 12 years, and did not cease to do so till she had attained her fiftieth year. Examined on the day of her entrance into the Hospital, the following was the report. 1mo. Slight pains were felt in the pelvis, and also in the loins—a sense of lassitude sufficiently distressing to prevent the perpendicular posture being long maintained. 2ndo. A discharge of sanious fluid, extremely fetid and sanguinolent, of about eight months' duration. 3tio. An examination shewed a loss of one of the lips of the os tincæ. The remaining one (the anterior) was thickened and ulcerated. The same alterations were perceptible in a certain portion of the vagina near the cervix uteri. This first examination did not induce any suspicion of adhesion between the parts affected and the rectum or bladder. On introducing the finger far up the rectum a tumour was found to occupy the situation of the uterus, and was concluded to be the diseased uterus itself. The various functions and the general health were in good condition. M. Recamier, who had once before extirpated the womb with success, determined on the operation in this case, and it was consented to without fear.

* *Clinique.*

On the 26th July, at 7 o'clock in the morning, the patient was placed on a bed in the midst of numerous pupils and others, besides Messrs. Margolain, Breschet, Palrice, and Blandin, and the operation was commenced. M. Recamier first introduced an instrument (pince de museux) with which he seized the cervix uteri as high up as possible, and then dragged it down slowly and cautiously, the body of the uterus following it, till the whole came in view from the os externum. The artificial prolapsus thus effected, the operator fixed the organ in its new situation by means of another instrument (pince erigne) confided to the hand of an assistant. He then examined the rectum, and found that the gut had not come down with the uterus. He next introduced his finger between the anterior parietes of the vagina and the corresponding face of the retracted tumour, till he came to the junction of the two, when he introduced a bistoury with his right hand, divided the cul-de-sac at the spot above-mentioned, to the extent of an inch, and then withdrew the instrument. Through this wound the operator passed his finger, and divided some loose cellular substance till he came to the reflection of the peritoneum from the bladder over the uterus. This he divided with the bistoury once more introduced. The peritoneal incision was next enlarged, first on one side, and then on another, with a probe-pointed bistoury. The operator having searched for the broad ligaments, he passed a ligature around each, to prevent hæmorrhage, and then divided them with the knife. The peritoneum passing from the rectum to the uterus was next divided, and the uterus itself dissected carefully away. The operation is said to have been conducted with remarkable sang froid,—to have occupied only twenty minutes, — and to have been succeeded by no hæmorrhage. The cervix uteri was found to be affected with open cancer; but the body of the uterus itself appears to have been very little affected. The patient bore the operation with unshaken courage, and fell asleep soon after she was put to bed. On the second day fever arose, and continued for two or three days, requiring several small bleedings. The fever ceased at the end of the fourth day, but was succeeded by a fixed pain in the right iliac region. Forty leeches were applied to this part, and the pain was reduced. The ligatures were removed on the fifth day, and on the sixth there was tension and swelling of the abdomen. Leeches were repeated. Nothing particular occurred till the ninth day, when, on examining the vagina, a fetid, dark, and sanguinolent fluid was found issuing forth. Injections were carefully thrown up during the next few days. By the twelfth day, when the account closes, she recovered some appetite, and had taken soup repeatedly. Should any thing further be published, we shall report it. Meantime the case may be considered as a successful one, as it is not now likely that the patient will die from the effects of the operation, which appears to have been performed with great skill and dexterity.

### II. SUPPOSED FOREIGN BODY IN THE EAR—HYDROCEPHALUS—TUMOUR IN THE RIGHT OPTIC THALAMUS.*

MR. PEARSON, we believe, used to say that if he *must* be deprived of one out of the two means of information in a case, the history or the symptoms actually present, he would hold to the former and reject the latter. We cannot conceive a more injudicious decision, for no one can guard against the misrepresentations or mistakes of his patient respecting antecedent events; whereas most men can perceive what is actually before their eyes. Our own conclusions are, that in very few cases should we place much reliance on the history *per se*, unless it be corroborated or confirmed by existing symptoms. This is our own impression, and we have now had some little experience in case-taking. The following instance will furnish a useful commentary on our position.

*Case*. A child, about 10 or 11 years

* Journ. Hebdom. No. 40.

of age, was brought by its parents to the Hôtel Dieu, with hemiplegia of the whole left side, and violent and painful retraction of the limbs on this side almost amounting to the tetanic spasm. On attempting extension of these parts the little patient struggled and screamed, and there was strabismus of the left eye. The parents declared that these symptoms had followed the introduction of the stone of a wild plum into the left ear, many years previously, which stone could never be extracted. On carefully examining the part M. Dupuytren could neither see nor find any foreign body, and the general cast of the symptoms led him to suspect organic disease of the brain. A seton was made in the nucha and light purgatives employed, apparently at first, with good effect. General convulsions, however, came on —the contraction of the muscles of the left side became more painful—the evacuations were passed involuntarily—and three months after its admission, the patient died.

*Dissection.* No disease of the bones at the basis cranii—*no foreign body whatever in the ear, which was sound.* The cerebral meninges were healthy, but the sub-arachnoid cellular membrane was infiltrated with serum, and much was also found in the lateral ventricles. In the substance of the right optic thalamus was a tumour the size of a small hen's egg, of rounded shape, reddish grey exterior, and marked with numerous rough points. Its consistence was firm and its texture homogeneous, of greenish colour, and filled with a number of small cavities containing serous fluid. The cavity in the cerebral matter in which the tumour lay was irregular, and its parietes were softened. The abdominal and thoracic viscera were sound.

Had dependence been placed on the relatives' account, or severe measures used to extract the foreign body affirmed to reside in the ear, and believed to produce the existing ills, what a blunder the surgeon would have made, what a risk the patient would have run! The case should teach us caution, especially when extraneous substances are merely said to be present in any of the outlets or inlets of the body, without tangible or other positive proof of the fact. The attempt to dislodge such imaginary obstructions not seldom excites unpleasant symptoms, and might readily give a fatal fill up to any that actually existed.

### III. Excessive Prolongations of the Mucous Membrane of the Upper Lip.

A young soldier in garrison at Paris presented himself in the Hôtel Dieu on the 20th of April, with a great prolongation of the mucous membrane of the upper lip, which almost entirely covered the teeth on each side when the patient attempted to speak or laugh. The double kind of pouch produced by this protrusion of the mucous membrane impeding the motions of the lip, independently of the disagreeable deformity it occasioned, the gallant soldier was very desirous of an operation for its removal. M. Dupuytren having directed an assistant to hold the upper lip on one side, seized it himself on the other, and nipped off the projecting mucous membrane with a pair of scissors, curved like those for excision of the tonsil on their flat side. A brisk hæmorrhage ensued, but was soon arrested; suppuration of the wound ensued, and at the end of eight days the patient was cured.

The reporter remarks, that the seat of this disease is in the sub-mucous cellular tissue, which is hypertrophied and more or less infiltrated with serum. The mucous glands of the lip, continually irritated by the suction, motions, and pinchings which the prolongation of the mucous membrane undergoes, are generally much enlarged also. In some individuals the permanent irritation induces chronic inflammation, which may even end in cancerous degeneration. In one case, M. Dupuytren was obliged to remove a great portion of the posterior part of the lip, which had become cancerous under these circumstances.

### IV. Remarks on Herniæ of the Muscles.

Be not alarmed, gentle student, we are not about to heave another Ossa on the Pelions and Olympus' that already intervene between you and your diploma. We are not to add another to the thousand and one varieties of hernia in and about that region so sacred to the divinities of surgery, the region of the groin. What we mean to describe is of quite another character.

After accidental openings, says M. Dupuytren, made in the fasciæ by surgical operations, wounds *not* surgical, or violent efforts, it frequently happens that the muscles during their contraction pass through the apertures, and form true hernial protrusions. They are sometimes very painful, and prevent the patients from using their limbs, and especially from walking, unless they are treated by appropriate bandaging. Occasionally the tumours thus formed give rise to strange errors in diagnosis, and consequent mischief in practice. A young man, for instance, the son of one of the members of the conseil général des hôpitaux, fatigued himself greatly in mounting a horse *à l'Anglaise,* whose manner of riding is well known to exercise greatly the muscles of the calf. A tumour supervened on the inner and posterior part of the leg, which was extremely painful on walking, or even standing still. When the patient lay down, the tumour disappeared and the pain subsided. Several practitioners were consulted on the case, some of whom thought it was a varix, others an enlargement of the nerves, but M. Dupuytren detected a protrusion of the muscles, such as has been mentioned above, and applied a bandage with complete success.

